# The potential contribution of impaired brain glucose metabolism to congenital Zika syndrome

**DOI:** 10.1111/joa.12959

**Published:** 2019-02-21

**Authors:** Javier Gilbert‐Jaramillo, Patricia Garcez, William James, Zoltán Molnár, Kieran Clarke

**Affiliations:** ^1^ Department of Physiology, Anatomy and Genetics University of Oxford Oxford UK; ^2^ Institute of Biomedical Sciences Federal University of Rio de Janeiro Rio de Janeiro Brazil; ^3^ Sir William Dunn School of Pathology University of Oxford Oxford UK

**Keywords:** congenital Zika syndrome, diet, glucose, ketone bodies, metabolism, microcephaly, Zika virus

## Abstract

The Zika virus (ZIKV) became a major worldwide public concern in 2015 due to the congenital syndrome which presents the highest risk during the first trimester of pregnancy and includes microcephaly and eye malformations. Several cellular, genetic and molecular studies have shown alterations in metabolic pathways, endoplasmic reticulum (ER) stress, immunity and dysregulation of RNA and energy metabolism both *in vivo* and *in vitro*. Here we summarise the main metabolic complications, with a particular focus on the possibility that brain energy metabolism is altered following ZIKV infection, contributing to developmental abnormalities. Brain energetic failure has been implicated in neurological conditions such as autism disorder and epilepsy, as well as in metabolic diseases with severe neurodevelopmental complications such as Glut‐1 deficiency syndrome. Therefore, these energetic alterations are of wide‐ranging interest as they might be directly implicated in congenital ZIKV syndrome. Data showing increased glycolysis during ZIKV infection, presumably required for viral replication, might support the idea that the virus can cause energetic stress in the developing brain cells. Consequences may include neuroinflammation, cell cycle dysregulation and cell death. Ketone bodies are non‐glycolytic brain fuels that are produced during neonatal life, starvation or fasting, ingestion of high‐fat low‐carbohydrate diets, and following supplementation with ketone esters. We propose that dietary ketones might alter the course of the disease and could even provide some degree of prevention of ZIKV‐associated abnormalities and potentially related neurological conditions characterised by brain glucose impairment.

## Introduction

Zika viruses (ZIKV), positive single‐stranded RNA [(+)ssRNA] mosquito‐transmitted viruses of the genus Flavivirus, were first isolated in 1947 from a febrile rhesus macaque in Uganda (Dick et al. 1952; Platt & Miner, 2017; Wen et al. 2017). Outbreaks in 2007 (Pacific islands), 2013 (French Polynesia; Cao‐Lormeau et al. 2014; Duffy et al. 2009; Musso et al. 2015) and 2015 (Brazil), were associated with an increase in birth abnormalities (Heymann et al. 2016; Wen et al. 2017) and other neurological disorders. Several studies have shown the propensity of ZIKV to infect neuronal tissue (Garcez et al. 2016; Petersen et al. 2016; Qian et al. 2016; Souza et al. 2016; Tang et al. 2016; Zhang et al. 2016; Bhatnagar et al. 2017; Li et al. 2017), hence associating it with neurological complications such as Guillain‐Barré syndrome, meningoencephalitis and myelitis (Blázquez & Saiz, 2016; Heymann et al. 2016; Pinheiro et al. 2016; Wen et al. 2017; Uncini et al. 2018).

Congenital microcephaly was principally observed when ZIKV infection occurred during the first and second trimesters of pregnancy (Cauchemez et al. 2016; Kleber de Oliveira et al. 2016; Pacheco et al. 2016), potentially due to vertical transmission through the placenta (Brasil et al. 2016; Calvet et al. 2016; de Noronha et al. 2016; de Paula Freitas et al. 2016; Ventura et al. 2016; Bhatnagar et al. 2017; Platt & Miner, 2017). ZIKV‐infection studies, both *in vitro* and *in vivo*, have shown that neuronal death, dysregulation of apoptosis and neurogenesis, and a decrease in brain size are common outcomes (Cugola et al. 2016; Rossi & Vasilakis, 2016; Tang et al. 2016; Garcez et al. 2017; Platt & Miner, 2017; Wen et al. 2017).

Brain reduction and microcephaly are phenotypes also exhibited by genetic mutations and/or energy impairment during neurodevelopment (Woods, 2004; Faheem et al. 2015). Microcephaly and Glut‐1 deficiency syndrome, where glucose uptake in the brain is decreased due to lack of expression of glucose receptors (Klepper et al. 2004; Jensen et al. 2006; Klepper, 2008; Tang et al. 2017), highlights the importance of energy supply for brain growth and function, as the brain is a sensitive organ with complex energy pathways and interactions (Owen et al. 1967; Dienel & Hertz, 2001; Cahill, 2006; Schönfeld & Reiser, 2013; Schousboe et al. 2014; Falkowska et al. 2015; Hofmann et al. 2017). In this review, we summarise data regarding the cellular processes exhibited during ZIKV infection and suggest that a potential cellular energetic failure underlies most of the cellular hallmarks, thus providing new insights for possible treatments.

## Energy metabolism in the developing and adult brain

### Brain fuels required for development and function

Neurons and glia have distinct requirements for energy substrates, particularly during activation (Schönfeld & Reiser, 2013; Tracey et al. 2018), yet the interactions between the metabolism of the different cell types remain unclear. To date, it has been hypothesised that the metabolism of neurons is more dependent on oxygen compared with glia metabolism (Itoh et al. 2003; Herrero‐Mendez et al. 2009).

**Figure 1:**
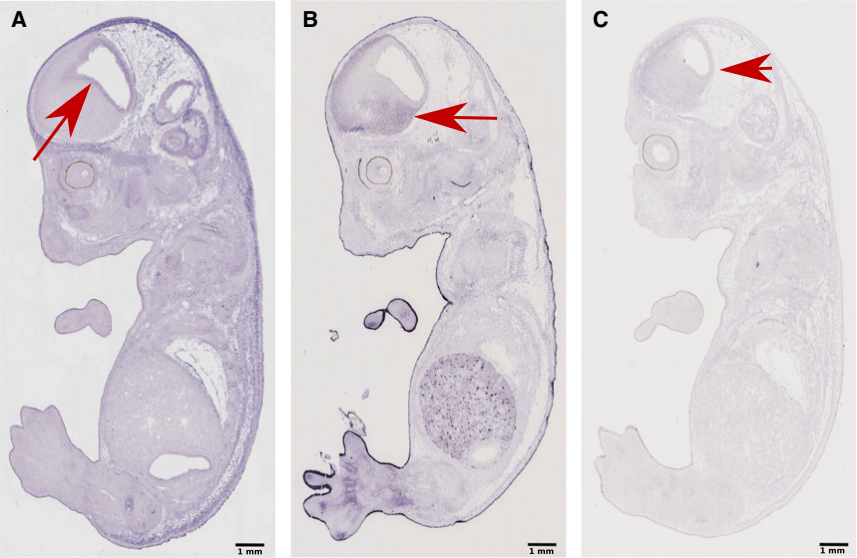
Glucose receptors in the embryonic mouse brain. genepaint sagittal plane sectioning image data of E14.5 Strain C57BL/6. (A) *In situ* hybridisation of the solute carrier family 2 (facilitated glucose transporter) member 1 (SLC2A1), mainly located in the ventricular zone at the neural progenitor region of the cerebral cortex and the ganglionic eminence (red arrow) (genepaint set ID: EH739). (B) *In situ* hybridisation of the solute carrier family 2 (facilitated glucose transporter) member 3 (SLC2A3) mainly located in postmitotic and differentiated neuronal populations in the primordial plexiform zone or preplate of the cortex and the striatum (red arrow) (genepaint set ID: EH4603). (C) *In situ* hybridisation of the solute carrier family 2 (facilitated glucose transporter) member 5 (SLC2A5) widely spread across the brain (red arrow) (genepaint set ID: EH4766). Scale bar: 1 mm.

Glucose is considered to be the primary fuel for the brain and is essential for normal placental and fetal growth (Hay, 2006). The glucose absorption for adequate brain development and activity (Devraj et al. 2011; Tang et al. 2017) is mainly mediated by glucose transporters (GLUTs), Glut‐1, Glut‐3 and Glut‐5, which are expressed across the placenta, blood‐brain barrier and neural progenitors, differentiated neuronal tissue, and microglia, respectively (Fig. 1) (Maher et al. 1991; Payne et al. 1997; Jurcovicova, 2014).

**Figure 2:**
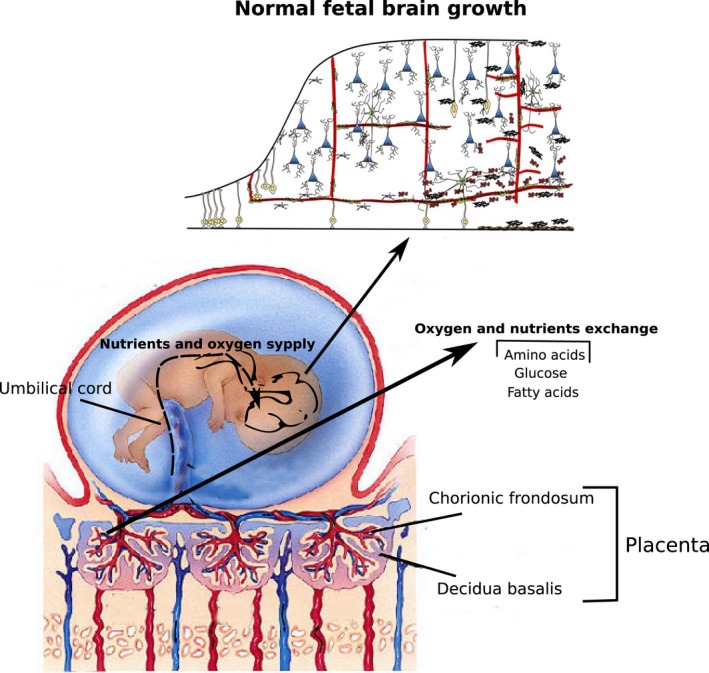
Nutrient delivery through the interactions of the maternal and fetal circulatory system for fetal growth. The maternal and fetal circulatory systems interact in the placenta to deliver the nutrients to the fetus. The main macro‐nutrients, glucose, amino acids and fatty acids, cross the placenta by means of their respective transporters (e.g. Glut‐1 and Glut‐3 for glucose molecules) and provide the energetic and structural requirements for normal fetal growth. During brain development, glucose is considered to provide energy for cell division and differentiation, amino acids contribute to the homeostasis and fatty acids are mainly used to generate myelin sheets during myelination. Modified from www.differencebetween.net (Copyright © The McGraw‐Hill Companies, Inc.), schoolbag.info and Stolp et al. (2012).

During fetal growth, the placenta regulates nutrient absorption (Fig. 2). In sheep, placental glucose uptake was directly correlated with the fetal artery concentration, independently of maternal plasma glucose concentration (Hay et al. 1990). Similarly, human studies showed that uteroplacental glucose absorption is exerted in both fetal and maternal sides of the placenta yet is slightly higher on the maternal side (Holme et al. 2015). Hence, transplacental glucose uptake is not exclusively related to the maternal glucose levels but is mostly determined by the fetal venous‐arterial glucose concentration (Hay et al. 1990; Schneider et al. 2003; Hay, 2006).

**Figure 3:**
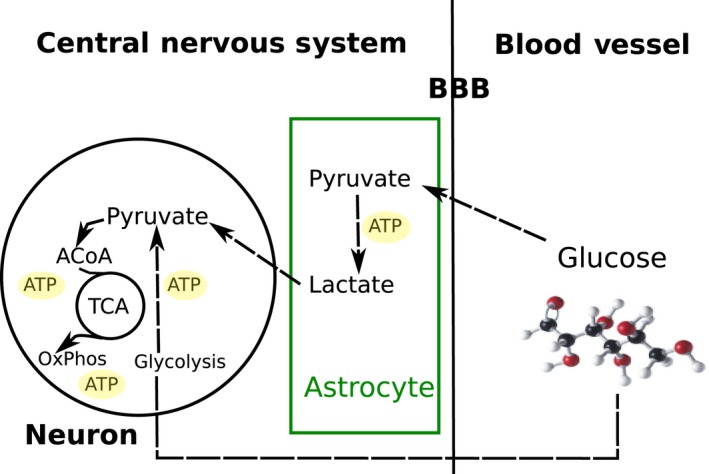
Glucose metabolism in astrocytes and neuronal cells. Glucose molecules available in the blood vessels cross the blood‐brain barrier (BBB) and are taken up by astrocytes and neurons. Astrocytes use aerobic glycolysis, despite the presence of oxygen, to produce ATP and Lactate (Warburg‐like effect). Neurons take up glucose directly from the bloodstream, which is converted to pyruvate via glycolysis. Lactate shuttled by astrocytes is also taken up by neurons and converted into pyruvate. Pyruvate inside the neuronal cytosol is converted into Acetyl‐CoA and enters the Krebs cycle, releasing by‐products for OxPhos to produce sufficient ATP, required for neuronal activity.

Neurons are able to convert glucose into Acetyl‐CoA for the production of substrates and the generation of ATP by oxidative phosphorylation, whereas glial cells, despite the presence of oxygen, preferentially convert glucose to lactate via cytosolic ‘aerobic glycolysis” (Schönfeld & Reiser, 2013; Camandola & Mattson, 2017); in a similar phenomenon to the ‘Warburg effect’ reported in oncology. Thus, it is hypothesised that the lactate produced by glial cells can also be metabolised by differentiated neurons for mitochondrial respiration and generation of ATP; potentially in a preferable way to glucose (Itoh et al. 2003). This complementary function of neurons and glia, known as the astrocyte‐neuron lactate shuttle (ANLS; Dienel & Hertz, 2001; Falkowska et al. 2015; Thevenet et al. 2016), potentially suggests that glial aerobic glycolysis may act as a fundamental mechanism to support neuronal metabolism (Fig. 3).

When exposed to the synaptic release of glutamate, astrocytes cycle glutamate/GABA to glutamine‐producing anaplerotic substrates that can feed and support the Krebs cycle maintenance (Dienel & Hertz, 2001; Schousboe et al. 2014) and hence oxidative phosphorylation. Astrocytes can also oxidise fatty acids to Acetyl‐CoA (Pellerin & Magistretti, 1994; Magistretti & Pellerin, 1999); therefore, upon activation such as during pre‐ and post‐synaptic processes, fatty acids metabolism in astrocytes is suggested to co‐occur together with cytosolic glucose oxidation to shuttle lactate for neuronal energy production while producing sufficient adenosine triphosphate (ATP) in the mitochondria to maintain the glutamate/glutamine cycle and astrocytic functions (Panov et al. 2014).

Moreover, oxygen is a limiting factor for brain activity. Higher oxygen consumption, particularly during synaptic activity, releases reactive oxygen species (ROS), to which brain cells are sensitive. Metabolism of ascorbic acid, the main brain antioxidant, is hypothesised to play a key role in the control of ROS toxicity (Castro et al. 2007, 2008). In brief, it has been suggested that ascorbic acid released from glial cells to the synaptic cleft and taken up by neurons, is oxidised to dehydroascorbic acid during ROS scavenging and released to be subsequently absorbed by astrocytes and re‐reduced to ascorbic acid to restart the cycle (Covarrubias‐Pinto et al. 2015).

**Figure 4:**
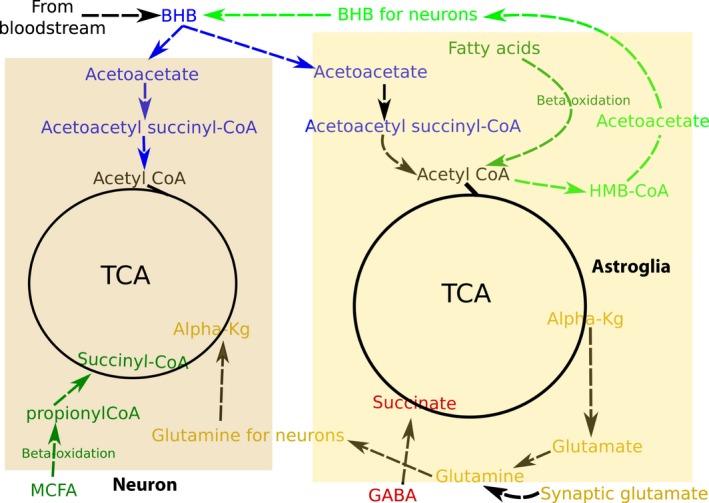
Model of non‐glycolytic metabolism interaction in astroglial and neuronal cells. In the presence of glucose, free fatty acids in the brain are taken up and oxidised by astroglial and neuronal cells to produce ATP via Krebs cycle/OxPhos as a supplementary energy source. Potentially, MCFA are mainly oxidised in neurons and other fatty acids in astroglial cells (dark green pathways). Under glucose deprivation: (1) astroglial cells can conduct ketogenesis to provide fast energetic substrates to neurons (light green pathway) and (2) ketone bodies from the bloodstream are taken up and oxidised by brain cells, providing up to ~70% of the energetic requirements (blue pathways). (3) Glutamate/glutamine is necessary to regulate the Krebs cycle, playing a major role in the communication of astroglia and neurons by processing excessive synaptic glutamate and providing amino acid glutamine. Also, but to a lesser extent, astroglial produced alpha‐ketoglutarate can be converted into glutamate to promote neurotransmitter and/or neuronal energetic by‐product release (mustard coloured pathways). (5). Neurotransmitter GABA can also be converted into a Krebs cycle metabolite depending on the needs of the mitochondrial Krebs cycle (red coloured pathway).

Because of the sensitivity of neurons to oxidative stress, it is expected that glial cells, rather than neurons, would oxidize fatty acids (Bélanger et al. 2011; Panov et al. 2014; Romano et al. 2017), yet medium‐chain fatty acids (MCFA) can be metabolised as an additional energy substrate in both glial cells and neurons (Augustin et al. 2018) (Fig. 4). Interestingly, in astrocytes, it has been shown that MCFA decrease the mitochondrial respiratory chain capacity without decreasing the intracellular levels of ATP, potentially by promoting an increase in aerobic glycolysis and/or ketogenesis (Thevenet et al. 2016).

Despite β‐oxidation of fatty acids providing a higher yield of ATP than glucose metabolism when considering the net ATP production per available carbon bond (7.1 mol ATP in palmitate compared with 6.4 in glucose), neuronal cells might not favour this pathway: (1) it requires more oxygen due to a higher number of Acetyl‐CoA molecules, (2) it produces higher levels of ROS during oxidative phosphorylation when NADH and FADH_2_ are oxidised, which has been associated with neuronal damage and death (Liu et al. 2017) and (3) the rate of ATP production from blood glucose matches the fast requirements of the neurons (Schönfeld & Reiser, 2013).

During neonate life, while breastfeeding, mammalians are exposed to a particular condition where carbohydrates, previously supplied by the maternal circulation, became restricted, and the diet is mainly constituted on fats (Bougneres et al. 1986; Gustafsson, 2009; Cotter et al. 2011). Ketone bodies, mainly synthesised in the liver when only low carbohydrates are available, are distributed, absorbed and utilised as an energy fuel in other organs, particularly the brain (Grabacka et al. 2016; Le Foll & Levin, 2016). Once carbohydrates become available in the diet, the previously described process can only occur under particular physiological stress conditions such as starvation, fasting and ingestion of high‐fat low‐carbohydrate diets. Ketone bodies in the brain, presumably exclusively produced in astrocytes to a lesser extent when high rates of free fatty acids are observed (Le Foll & Levin, 2016), are mainly diffused from the bloodstream and account as the major substrate to provide energy (~ 70%) under glucose deprivation (Fig. 4) (Owen et al. 1967; Morris, 2005; Cahill, 2006; Owen, 2006; Klepper, 2008).

Ketone body metabolism is commonly studied based on the most abundant blood ketone body, β‐hydroxybutyrate (BHB), but two other ketone bodies (acetoacetate and acetone) are produced as result of ketogenesis. While BHB and acetoacetate are used by the cells to produce energy, acetone is released when breathing, serving as an indicator of the ketotic state (Musa‐Veloso et al. 2002). As energy substrate, BHB is converted into acetoacetate by 3‐hydroxybutyrate dehydrogenase; acetoacetate molecules are converted to acetoacetyl‐CoA by acetoacetyl succinyl CoA transferase to produce two molecules of acetyl‐CoA through the effects of acetoacetyl‐CoA thiolase. Molecules of acetyl‐CoA enter the Krebs cycle and respiratory chain to produce ATP (Grabacka et al. 2016).

## ZIKV emergence and importance

The worldwide emergence of ZIKV became a major public health concern in 2015 when the epidemic in Brazil was shown to be highly correlated with an increase in abnormalities in newborn children (Musso et al. 2015; Heymann et al. 2016; Coelho & Crovella, 2017). Brazil was the first country in the American continent to suffer from the ZIKV epidemic, but it is now widespread on the entire continent (Pacheco et al. 2016; Colón‐González et al. 2017), with the potential of compromising currently unaffected countries where there is no herd immunity and where the environmental conditions allow the reproduction of mosquitos and viral transmission (Messina et al. 2016; Samy et al. 2016; Colón‐González et al. 2017).

The public concern about ZIKV disease and the neurological complications (Araujo et al. 2016; Blázquez & Saiz, 2016; Rabaan et al. 2017) relies heavily on the potential routes of transmission and infection (Gebre et al. 2016). ZIKV is mainly transmitted by infected *Aedes* mosquitos, sexual relations and vertical (fetal‐placental) transmission (Araujo et al. 2016; Brasil et al. 2016; Calvet et al. 2016; Cugola et al. 2016; Kleber de Oliveira et al. 2016; Bhatnagar et al. 2017; Weaver, 2017; Shi et al. 2018), and, according to other research, it may potentially be transmitted by blood transfusions and breastfeeding (Gebre et al. 2016; Colt et al. 2017; Deng et al. 2017; Sharma & Lal, 2017).

The complications due to ZIKV infections are variable, being either asymptomatic (~ 80% of the cases) or symptomatic (Gebre et al. 2016; Rabaan et al. 2017). Symptomatic infections exhibit a generalised mild fever, myalgia, arthralgia and headache, barely distinguishable from other diseases such as influenza. However, additional symptoms can include rash and conjunctivitis (Duffy et al. 2009; Hayes, 2009; Chen & Hamer, 2016; Rabaan et al. 2017), similar to those seen with other flaviviruses such as dengue (DENV) and chikungunya viruses (Mlakar et al. 2016). As both prevention of infection and early and specific diagnosis are challenging, it is highly desirable to develop post‐infection treatments to prevent risks to pregnancy, birth defects and adult complications (Krow‐Lucal et al. 2018).

## ZIKV infection: fetal abnormalities and potential long‐term neurological effects

The Zika virus has been shown to infect different tissues such as *decidua*, fetal placenta and the umbilical cord (El Costa et al. 2016) which support vertical transmission; however, the virus has also shown a marked preference for neuronal tissue (Garcez et al. 2016; Petersen et al. 2016; Qian et al. 2016; Souza et al. 2016; Tang et al. 2016; Zhang et al. 2016; Bhatnagar et al. 2017; Li et al. 2017). It has been shown that ZIKV‐related fetal neurological complications are highest during the first trimester of pregnancy (El Costa et al. 2016; Bhatnagar et al. 2017; Honein et al. 2017). However, findings suggest that these may also continue to occur during the second (Lin et al. 2017) and third trimesters (Hayes, 2009; Shapiro‐Mendoza, 2017) to a lesser extent (Bhatnagar et al. 2017).

The Zika virus preferential infection for neural stem cells and progenitors is more marked than for other flaviviruses (Brault et al. 2016; El Costa et al. 2016; Miner & Diamond, 2017; Ming et al. 2016; de Noronha et al. 2016; Martines et al. 2016; Souza et al. 2016; Zhang et al. 2016), potentially explaining why adult symptomatology is mostly mild with no further complications but causing severe neurological complications in the fetus and newborns (Brault et al. 2016; Hughes et al. 2016).

Among the ZIKV fetal/newborn complications, a reduction in the head and brain size, called microcephaly, together with eye malformations are the most relevant abnormalities (Araujo et al. 2016; Mlakar et al. 2016; Sarno et al. 2016; Merfeld et al. 2017). However, the infection can also be the cause of long‐term mental health conditions such as cognitive impairment, behavioural and neurological complications in newborns with no evident phenotype after maternal immune activation due to viral, including flaviviral, and bacterial infections (Verma et al. 2011; Stolp et al. 2012; Estes & McAllister, 2016; Lombardo et al. 2018).

### Microcephaly and eye malformations

Microcephaly in newborns is characterised by a head circumference of 2 SD smaller than the local average. Microcephaly can vary broadly in clinical severity with differences in the brain mass morphology (Woods, 2004; Adachi et al. 2014), underlying severe neuronal loss in the cerebral cortex (Azevedo et al. 2009; Merfeld et al. 2017) and/or reduction in the population of glial cells (Lin et al. 2017). Microcephaly is characterised by abnormalities in the production/differentiation of neural progenitor cells (NPC; Gilmore & Walsh, 2013) potentially through alterations in the endothelial cells of the brain blood vessels that directly alter NPC homeostasis by decreasing nutrients flow and trophic factors (Shen et al. 2004; Garcez et al. 2018). Primary microcephaly is the disease type caused by diverse genetic and epigenetic factors, including maternal viral infections (Woods, 2004; Faheem et al. 2015; Zhang et al. 2016; Merfeld et al. 2017) with an estimated rate of 2–12 cases per 10 000 births which increased up to ~ 20‐fold in early 2016 in Brazil due to ZIKV infections (Duffy et al. 2009; Cao‐Lormeau et al. 2014; Heymann et al. 2016; Mlakar et al. 2016; Coelho & Crovella, 2017; Cunha et al. 2017; Krow‐Lucal et al. 2018), with fewer neurological complications in other countries (Pacheco et al. 2016; Colón‐González et al. 2017; Honein et al. 2017; Rick et al. 2017).

ZIKV‐related microcephaly has been intensively studied since the outbreak in Brazil, showing negative effects on neurogenesis (Wen et al. 2017) and microgliogenesis (Li et al. 2018). Microgliogenesis is a process that occurs alongside neurogenesis and is of high relevance during development, as a close interaction between microglia and neuronal cells is maintained in the developing and adult CNS in both health and disease (Nayak, Roth, & McGavern, 2014), potentially suggesting a major role of microglia activation/death in ZIKV‐microcephaly.

As previously stated, cell and animal model studies of ZIKV‐microcephaly have been widely investigated to reveal the potential causes of the neurological complications showing dysregulation of different genes related to neuronal differentiation, neuronal growth, cellular metabolic function and cell death (Tang et al. 2016; Zhang et al. 2016; Devhare et al. 2017; Garcez et al. 2017; Lin et al. 2017), providing insights into potential prevention mechanisms.

The Zika virus congenital eye malformations are yet to be fully determined due to the poor knowledge of the full spectrum of ocular lesions (Jampol & Goldstein, 2016; de Paula Freitas et al. 2017). Nevertheless, reports of infants’ studies exhibited focal pigment mottling of the retina, atrophy in the chorioretina, alteration in the retinal vasculature, optic nerve abnormalities, bilateral iris coloboma and lens subluxation (de Paula Freitas et al. 2016; Garcez et al. 2018) and, in the fetus, loss of retinal epithelium, thin choroid, perivascular choroidal inflammatory infiltrate and atrophy of the optic nerve (Fernandez et al. 2017).

## Cellular changes, damage and death in the CNS during ZIKV infection

### ZIKV compromises neuronal and glial cell homeostasis

Recent research focused on the cellular and molecular dysregulation during ZIKV infections has provided insights into different potential biological processes responsible for neurological damage and microcephaly (Tang et al. 2016; Merfeld et al. 2017).

In a similar fashion to DENV (Savidis et al. 2016), ZIKV replication might activate early glycolysis and late fatty acids β‐oxidation; inducing a change in lipid metabolism. Observed lipid droplet formation, autophagy (Samsa et al. 2009; McLean et al. 2011; Tiwari et al. 2017; Tongluan et al. 2017; Lee et al. 2018) and enhanced higher intracellular ATP levels (Samsa et al. 2009; Heaton & Randall, 2010, 2011; Vidali et al. 2015) could be supported by lipid β‐oxidation processes.

**Figure 5:**
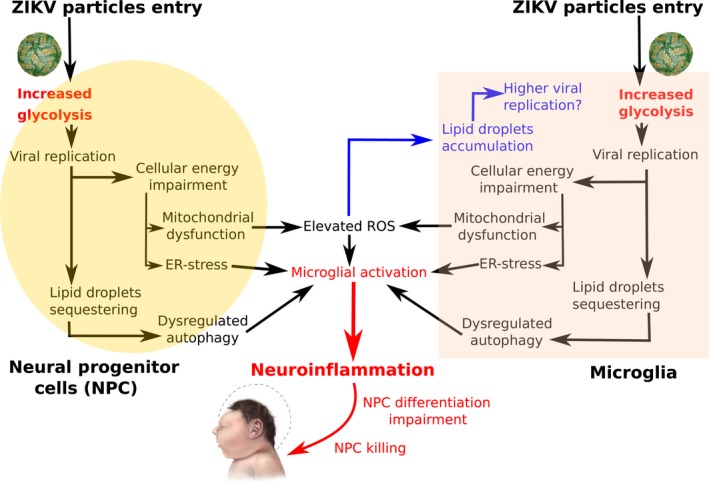
Hypothesised key role of glycolysis and neuroinflammation in congenital ZIKV syndrome. Increased glycolysis following ZIKV infection and replication in the endoplasmic reticulum (ER) of both neural progenitor (NPC) and microglial cells potentially causes (1) cellular energy impairment with further mitochondrial dysfunction and ER stress, and (2) sequestration of cytoplasmic lipid droplets required for final replication (pathways highlighted in black). Elevated reactive oxygen species (ROS) due to mitochondrial dysfunction cause an accumulation of lipid droplets in microglia, potentially favouring a higher viral replication rate than in neurons (pathway highlighted in blue). (3) Microglial activation by dysregulated autophagy, ER stress and elevated ROS contribute to neuroinflammation, impairing NPC differentiation and promoting NPC killing mechanisms (pathway highlighted in red); a possible explanation for brain growth impairment and congenital ZIKV syndrome.

Of relevance, ZIKV infection causes ER stress (Blázquez et al. 2014; Gladwyn‐Ng et al. 2018) and cell death (Fig. 5). ER stress occurs when an accumulation of unfolded proteins causes perturbation of the ER (Hetz, 2012; Cornejo et al. 2013). In response to this, a mechanism mainly conducted by three proteins named inositol‐required enzyme 1 (IRE1α), protein kinase RNA‐like ER kinase (PERK) and activating transcription factor 6 (ATF6) potentially alleviates the stress via decreasing mRNA translation and/or by degradation of unfolded proteins (Hetz, 2012; Cornejo et al. 2013; Sano & Reed, 2013).

In a normal cellular state, PERK and ATF6 remain inactive and bound to the ER, while IRE1α appears to be activated only when unfolded proteins bind to it (Gardner & Walter, 2011). Interestingly, PERK is upregulated in the brains of ZIKV‐infected humans, and this is replicated in both murine models and human cultured neural stem cells challenged with ZIKV in which downregulation of disturbed PERK pathway prevents microcephaly, potentially highlighting mechanisms for therapy (Gladwyn‐Ng et al. 2018).

In most viral infections, the innate immune system is highly responsible for countering viral replication (Xie et al. 2016). Interferon (IFN) activation in response to infection plays a key part of this response that restricts virus replication, depending on the viral strain (Evans et al. 2011; Lubick et al. 2015; Xia et al. 2016). ZIKV infection particularly alters the IFNAR1, by showing a decrease on the mRNA expression levels (Tang et al. 2016; Tiwari et al. 2017); suggesting a diminished innate immune response, potentially favouring viral replication.

To this end, several *in vivo* murine models systems of *Ifnar1* are used to study ZIKV disease phenotypes such as microcephaly and ocular abnormalities (Bayer et al. 2016; Cugola et al. 2016; Morrison & Diamond, 2017), yet the inoculation route and mice strains should be considered. For example, when infecting ^–^C57BL/6 mice subcutaneously, intravenously or intraperitoneally, weight loss, paralysis and high rates of mortality (20–100%) are observed; however, intrauterine growth impairment is not (Lazear et al. 2016; Ma et al. 2016; Miner et al. 2016), and when infecting subcutaneously on Ifnar1^−/−^ mated with WT C57BL/6, the common phenotypes are placental injury, resorption and intrauterine growth impairment (Miner et al. 2016; Sapparapu et al. 2016; Yockey et al. 2016; Morrison & Diamond, 2017).

Furthermore, evidence of a major role for microglia‐mediated neuroinflammation in ZIKV‐associated phenotypes (Fig. 5) includes the observation that ZIKV infection of microglia inhibits the differentiation of neural precursors (Mesci et al. 2018; Wang et al. 2018) and leads to the death of damaged neurons (Brown & Vilalta, 2015). The downregulation of immunological genes in ZIKV‐infected microglia (Tiwari et al. 2017) suggests that the virus has evolved sophisticated strategies for modulating the innate antiviral response.

### Glycolytic and cellular energetic dysregulation during ZIKV

Edward Blonz hypothesised that, similar to DENV and other viruses, ZIKV replication might alter brain cell glucose uptake by close interaction with the Glut‐1 receptor (Yu et al. 2011a,b; Fontaine et al. 2014). In this setting, brain energetic failure could potentially be the cause of ZIKV congenital malformations, as similar phenotypes are exhibited in Glut‐1 deficiency syndrome (Jensen et al. 2006; Blonz, 2016; Solomon et al. 2016; Tang et al. 2017).

In this context, research has shown that, as with DENV (Fontaine et al. 2014; Jordan & Randall, 2016; Rothan et al. 2018), glycolysis is used to produce energy, potentially for optimal ZIKV replication (Tiwari et al. 2017). Increased glycolysis evokes cellular energy impairment, which contributes to ER stress via the PERK pathway (de la Cadena et al. 2014), mitochondrial dysfunction and ROS production, all contributing to neurodegeneration (Rossignol & Frye, 2012; Liu et al. 2017).

Moreover, elevated ROS production in the brain contributes to lipid droplet accumulation, particularly in glial cells (Liu et al. 2015). Lipid droplets are intracellular storages with a crucial role in fatty acid trafficking and energy homeostasis, while also being a source of lipid metabolism in viral immunity (Welte, 2015). ZIKV, similar to DENV, potentially triggers lipid droplet accumulation for (1) final replication of the viral capsid and (2) increasing ATP availability in the cell via beta‐oxidation of fatty acids (Samsa et al. 2009; Heaton & Randall, 2010, 2011), causing dysregulated autophagy and, together with elevated ROS and ER stress, neuroinflammation, potentially explaining neural progenitors death, increased blood‐brain barrier permeability and brain growth impairment (Fig. 5).

Despite the higher complexity in brain energetics, which might be specific to brain regions, cellular subtypes and activity, glucose metabolism plays a major role as the main energetic substrate, and protein degradation and fatty acid oxidation are hypothesised to be conducted principally in glial cells (Dienel & Hertz, 2001; Panov et al. 2014; Schousboe et al. 2014; Romano et al. 2017). For these reasons, glucose metabolism could be implicated as the potential main cause for congenital ZIKV syndrome.

Geographical distribution can play a role in congenital ZIKV syndrome, with the highest prevalence of the epidemic being observed in the North‐East region of Brazil, a population characterised by poverty (Jimena Barbeito et al. 2018bb; Krow‐Lucal et al. 2018), presenting with comorbidities such as malnutrition. Therefore, this review considers that specific dietary patterns favouring the activation of certain metabolic pathways might play an important role in the susceptibility or protection against ZIKV virulence (Jimena Barbeito et al. 2018a).

## Potential nutritional intervention to prevent ZIKV replication and fetal abnormalities

### Ketones induce positive effects in brain homeostasis under glucose deprivation

Energetic deficit due to glucose impairment has a direct negative impact on human brain development and is therefore of potential consideration in ZIKV infection, particularly during the first trimester of pregnancy. As Blonz (2016) hypothesised, a high‐fat metabolism might prevent congenital abnormalities due to glucose impairment. Here we highlighted a potential link between cellular glucose impairment and different cellular alterations exhibited during ZIKV infection, emphasising that a ketone metabolism, as an efficient brain alternative fuel with positive effects in human physiology (Klepper et al. 2005; Morris, 2005; Owen Oliver, 2006), might prevent ZIKV‐related congenital phenotypes.

In addition to the positive effects of ketone metabolism in restoring glucose energetic impairment (Owen, 2006), ketones have been shown: (1) to contribute to brain homeostasis under glucose deprivation by reducing neuroinflammation via the restoration of mitochondria energetics (Vidali et al. 2015) and regulating autophagic flux to prevent neuronal death (Camberos‐Luna et al. 2016); (2) to decrease ROS release (Maalouf et al. 2007; Kim et al. 2010); (3) to alleviate energetic failure‐related ER stress (Bae et al. 2016; Soejima et al. 2018); (4) to promote efficient immune response (Kono et al., 2004; Rhyu & Cho, 2014).

The above raises the possibility that ketone metabolism, by either ingestion of a ketogenic diet or ketone ester supplementation, might prevent ZIKV‐congenital neurological phenotypes (1) by an energetic metabolic switch potentially preventing/decreasing ZIKV replication, via blocking glycolysis (Randle, 1998; Hue & Taegtmeyer, 2009), if it is essential for viral replication, and/or (2) by providing additional fuel to regulate cell functioning and homeostasis, promoting an efficient brain immune response against ZIKV infection. Finally, it is relevant to highlight that the neurological benefits of a ketone metabolism need to be further investigated in ZIKV infection, as it might ameliorate related adult mental/cognitive conditions and potential late‐onset complications in cases with no exhibited congenital syndrome.

## Declaration of interest

The authors declare no conflicts of interest and that no interests of any person/organisation are affected by the information presented in the present manuscript. J.G.‐J. held a scholarship from the Ecuadorian National Government. The Molnár Laboratory held an MRC Rapid Response Grant in collaboration with Dr Patricia Garcéz (Federal University of Rio de Janeiro, Brazil).
